# Lower-Limb Amputees Adjust Quiet Stance in Response to Manipulations of Plantar Sensation

**DOI:** 10.3389/fnins.2021.611926

**Published:** 2021-02-18

**Authors:** Courtney E. Shell, Breanne P. Christie, Paul D. Marasco, Hamid Charkhkar, Ronald J. Triolo

**Affiliations:** ^1^Department of Biomedical Engineering, Lerner Research Institute, Cleveland Clinic, Cleveland, OH, United States; ^2^Advanced Platform Technology Center, Louis Stokes Cleveland VA Medical Center, Cleveland, OH, United States; ^3^Department of Biomedical Engineering, Case Western Reserve University, Cleveland, OH, United States; ^4^Research and Exploratory Development Department, Johns Hopkins University Applied Physics Laboratory, Laurel, MD, United States

**Keywords:** transtibial amputation, peripheral nerve stimulation, sensory feedback, standing balance, vibration, neuroprostheses, somatosensation, balance perturbation

## Abstract

Interfering with or temporarily eliminating foot-sole tactile sensations causes postural adjustments. Furthermore, individuals with impaired or missing foot-sole sensation, such as lower-limb amputees, exhibit greater postural instability than those with intact sensation. Our group has developed a method of providing tactile feedback sensations projected to the missing foot of lower-limb amputees via electrical peripheral nerve stimulation (PNS) using implanted nerve cuff electrodes. As a step toward effective implementation of the system in rehabilitation and everyday use, we compared postural adjustments made in response to tactile sensations on the missing foot elicited by our system, vibration on the intact foot-sole, and a control condition in which no additional sensory input was applied. Three transtibial amputees with at least a year of experience with tactile sensations provided by our PNS system participated in the study. Participants stood quietly with their eyes closed on their everyday prosthesis while electrically elicited, vibratory, or no additional sensory input was administered for 20 s. Early and steady-state postural adjustments were quantified by center of pressure location, path length, and average angle over the course of each trial. Electrically elicited tactile sensations and vibration both caused shifts in center of pressure location compared to the control condition. Initial (first 3 s) shifts in center of pressure location with electrically elicited or vibratory sensory inputs often differed from shifts measured over the full 20 s trial. Over the full trial, participants generally shifted toward the foot receiving additional sensory input, regardless of stimulation type. Similarities between responses to electrically elicited tactile sensations projected to the missing foot and responses to vibration in analogous regions on the intact foot suggest that the motor control system treats electrically elicited tactile inputs similarly to native tactile inputs. The ability of electrically elicited tactile inputs to cause postural adjustments suggests that these inputs are incorporated into sensorimotor control, despite arising from artificial nerve stimulation. These results are encouraging for application of neural stimulation in restoring missing sensory feedback after limb loss and suggest PNS could provide an alternate method to perturb foot-sole tactile information for investigating integration of tactile feedback with other sensory modalities.

## Introduction

Maintaining balance during quiet stance requires constant coordination between motor commands and sensory feedback. Muscles activate to keep the body’s center of mass located over the base of support, and commands to involved musculature are updated based on visual, vestibular, and somatosensory inputs (Forbes et al., 2018; MacKinnon, 2018). Motor commands, sensory feedback, and coordination between the two can be interrupted in a variety of ways. External perturbations come from modifications to the environment, such as changes in the surface of the ground or being pushed (Dickin and Doan, 2008; Rogers and Mille, 2018). Internal perturbations arise from the body itself, such as muscle fatigue, closing the eyes, or planned movements (Dickin and Doan, 2008; Rogers and Mille, 2018). When perturbations are unexpected, the motor control system uses sensory feedback to detect the perturbation and makes adjustments in response to what is detected (Forbes et al., 2018; MacKinnon, 2018). The nature of these responses provides insight about how sensory input is incorporated into the body’s control scheme, and speaks to the meaningfulness and utility of the feedback.

Tactile inputs from the sole of the foot provide feedback about foot-floor contact, including center of pressure and its location relative to the base of support (Strzalkowski et al., 2018; Viseux et al., 2019). This information helps the motor control system determine support surface characteristics, detect changes in foot-floor interactions, and, when integrated with other sensory inputs, define body orientation (Maurer et al., 2001; Chien et al., 2014, 2016; Ku et al., 2014). In quiet stance, plantar sensory feedback contributes to stability and control of small-amplitude body sway (Kavounoudias et al., 1998, 1999). Individuals with deficiencies in cutaneous plantar sensation typically exhibit postural instability (e.g., Lord et al., 1991; Simoneau et al., 1994; Boucher et al., 1995; Uccioli et al., 1995; Hughes et al., 1996). For example, lower-limb amputees have greater variations in their center of pressure location and rely more on vision than able-bodied individuals, due in part to impaired feedback about foot-floor interactions (for review, Ku et al., 2014).

We have developed a peripheral nerve stimulation (PNS) system that can provide tactile sensations that are projected to the missing foot of lower-limb amputees. In place of the missing biological mechanoreceptors, this system provides specific tactile sensations that can be modulated by changing the stimulation delivered. Electrical current delivered to the nerves via individual electrode contacts of a high-density nerve cuff can be selectively tuned to elicit sensations of touch and pressure at discrete areas on the missing foot (Charkhkar et al., 2018, 2020; Christie et al., 2020). Our findings along with other reports suggest that activating remaining neural pathways in the residual limb of lower-limb amputees can provide sensory feedback from the missing limb that informs user behavior (Clites et al., 2018; Petrini et al., 2019b; Charkhkar et al., 2020). Modulating evoked tactile sensations in response to foot-floor contact pressures enhances balance control during perturbations in the visual field and stance surface (Charkhkar et al., 2020) and improves the tradeoff between speed and accuracy in an ambulatory searching task (Christie et al., 2020). It can also decrease the number of falls when walking over obstacles (Petrini et al., 2019b). However, it is still unclear how sensorimotor control is impacted by foot-sole tactile sensations induced by PNS. Providing insight into the way the nervous system processes electrically elicited sensations is necessary to optimize future interventions and more effectively integrate them with the resources remaining after limb loss.

Previous studies have explored the role of foot-sole tactile sensation in sensorimotor control by using various methods to perturb foot sole cutaneous feedback in able-bodied individuals. Disruptions or modifications limited to specific areas of the foot cause real and perceived directional postural adjustments. Indenting the skin of the foot sole with a matrix of pins or small metal pellets causes postural adjustments (Watanabe and Okubo, 1981; Maurer et al., 2001), as does use of textured insoles (Corbin et al., 2007; Qiu et al., 2012). When free to move, able-bodied individuals lean away from areas of the foot that are vibrated (Kavounoudias et al., 1998) or iced (Nurse and Nigg, 2001). When the body is prevented from swaying, opposite effects are observed: participants reported feeling as though they leaned toward the applied vibration (Roll et al., 2002). If the nervous system interprets plantar sensations elicited by PNS in a similar fashion, we would expect to observe similar responses in shifts and variation of center of pressure and posture. Thus, vibration on the intact foot sole can provide a reference for sensorimotor system responses to changes in tactile inputs.

During static standing with the eyes closed, we examined postural adjustments to randomly timed internal perturbations from PNS-induced tactile sensations perceived as originating on the missing foot sole. We compared these adjustments to responses to vibration on the intact foot sole and to static standing with no neural or vibratory stimulation. Our primary hypothesis was that PNS in the amputated limb would cause similar postural responses as vibration of the intact limb. The ability of PNS to perturb stance in a manner comparable to mechanical vibration would indicate that the sensorimotor control system incorporates electrically elicited sensations in an analogous way, and suggest that PNS could provide tactile sensations that are physiologically relevant. Thus, these experiments represent a step toward understanding how PNS-induced tactile feedback can be better used to improve postural control for lower-limb amputees. This work also explores a novel way of providing somatosensory perturbations during a task without changing the physical environment or directly modifying biomechanics.

## Materials and Methods

### Participants

**FIGURE 1:**
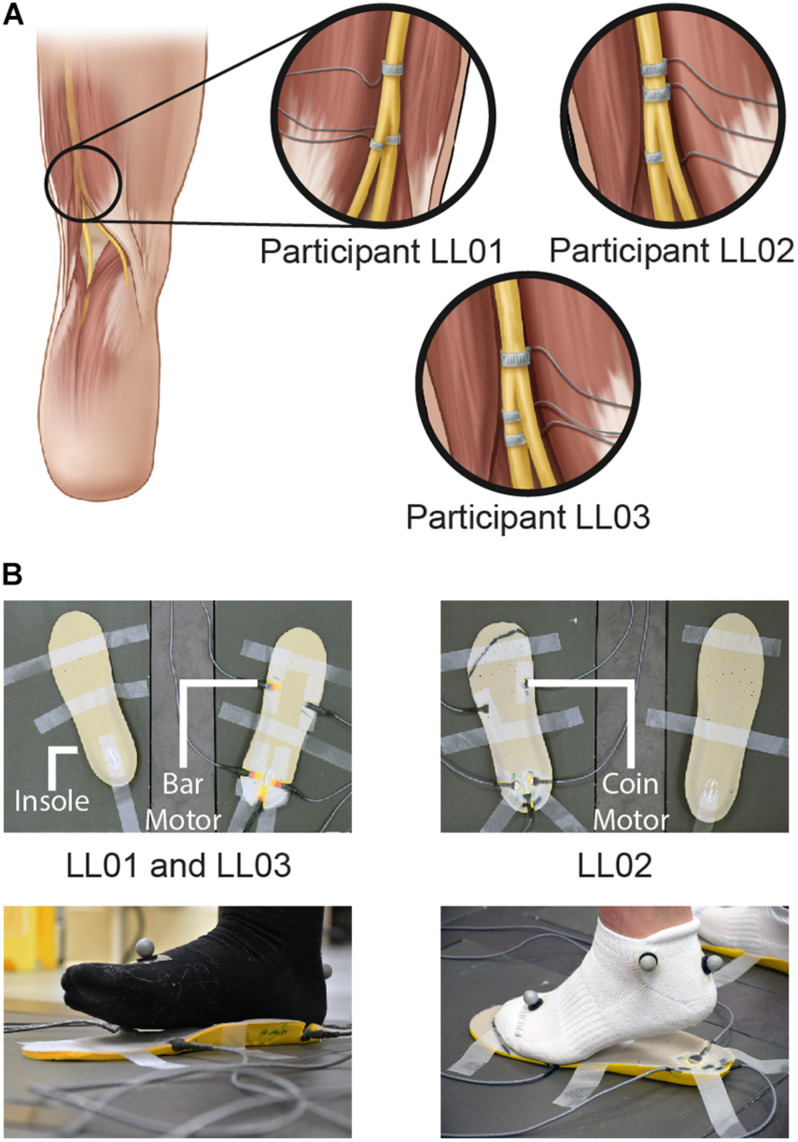
Methods of delivering unilateral tactile stimuli. **(A)** Tactile percepts were elicited in the missing foot via peripheral nerve stimulation (PNS). Electrical stimulation was delivered through cuff electrodes implanted around the sciatic nerve and its branches. **(B)** Vibration was delivered to the intact foot via vibrating motors incorporated into an insole. Vibrating bar motors were used for participants LL01 and LL03, and vibrating coin motors were used for participant LL02. Illustrations in 1A are provided courtesy of the APT Center at the Louis Stokes Cleveland VA Medical Center.

Three volunteers who had previously undergone transtibial amputation (Table 1) were implanted with 16-contact composite flat interface nerve electrodes (C-FINEs) around the sciatic, tibial, and/or common peroneal nerves in their residual limb (Figure 1A). All contacts were accessible by an external stimulator via percutaneous leads exiting the skin on the upper anterior thigh. Additional details of the surgical procedure and implanted technology are described elsewhere (Charkhkar et al., 2018). Prior to performing these experiments, participants had at least 1 year of experience with stimulation through the nerve cuff electrodes and perceived plantar sensations at discrete and repeatable locations in response to stimulation. The Louis Stokes Cleveland Veterans Affairs Medical Center Institutional Review Board and Department of the Navy Human Research Protection Program approved all procedures. This study was conducted under an Investigational Device Exemption obtained from the United States Food and Drug Administration. All participants gave their written informed consent to participate in this study.

**TABLE 1:** Participant characteristics.

**ID**	**Gender**	**Amputation etiology**	**Amputated limb**	**Age (years)**	**Mass (kg)**	**Years since C-FINE implantation**
LL01	M	Trauma	Left	70	104	2.9
LL02	M	Trauma	Right	56	67	2.4
LL03	M	Trauma with a non-healing wound that led to amputation	Right	68	89	1.3

### Tactile Stimuli

Participants received open-loop sensory stimuli either through the nerve cuff electrodes implanted in their residual limb or insole-mounted vibration units under their intact limb. For PNS in each participant, we selected two electrode contacts and stimulation parameters that consistently produced distinct sensations on the missing foot sole, described as tingling, pulsating, or pressure from toes curling. Charge-balanced, monopolar, asymmetric biphasic, cathodic-first pulses were delivered to single C-FINE contacts and a common, skin-mounted return electrode was placed at the iliac crest. Stimulation was delivered by an external stimulator controlled in real time via MATLAB (MathWorks, Inc., Natick, MA, United States) (Charkhkar et al., 2018). At the start of the experiment, stimulation parameters were tuned by manipulating pulse amplitude, width, and frequency to provide a range of comfortable, perceptible sensations. The parameters varied by contact, but the same parameters were used in all trials for a given condition and participant. Pulse amplitude ranged from 0.8 to 1.2 mA, pulse width ranged from 120 to 240 μs, and pulse frequency was set at either 20 or 100 Hz. Previous work has shown that the delay between stimulation onset and perception of stimulation-induced sensation for this system is not significantly different from natural tactile sensation and perceived faster than a visual stimulus (Christie et al., 2019).

For the intact side, five vibrating motors were inset into an insole (HappyStep Regular Fit Memory Foam Insole, size 8–12, Universal Electrical Supply Ltd., Toronto, Canada) under the intact foot at the first and fifth metatarsals and under the heel (Figure 1B). The complementary insole was placed under the prosthetic foot without vibrating motors to ensure comparable leg lengths. All vibrating motors in a selected region (forefoot or rearfoot) were activated simultaneously and vibration was controlled in real time by MATLAB. One participant, LL02, had a lighter mass than the other two participants and found the intensity of the vibration to be much higher than that of the PNS. To maintain similarity in perceived stimulus intensity between the affected and intact sides, the lighter participant stood on coin motors (220 Hz, Model C1234B016F, Kysan Electronics, San Jose, CA) while the heavier participants stood on bar motors (190 Hz, Model 307-103, Precision Microdrives, London, United Kingdom).

**FIGURE 2:**
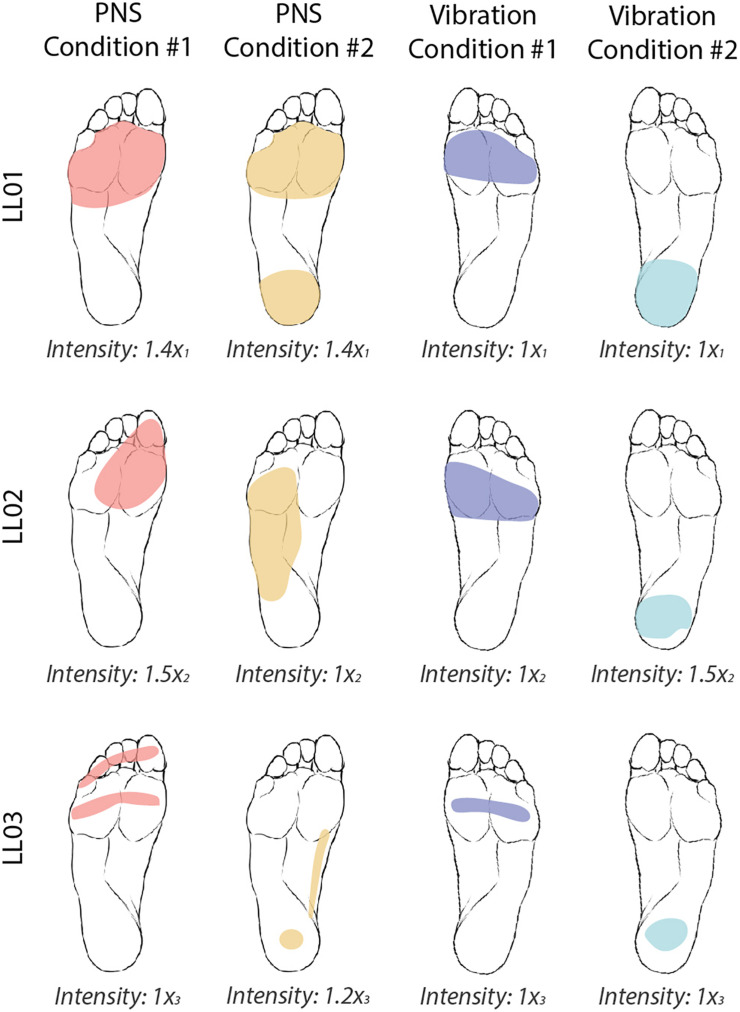
Locations and intensities of tactile stimuli reported by participants. The participants were asked to draw the locations of the four tactile stimuli on an image of a healthy foot. PNS-evoked percepts in the missing foot are shaded in red and yellow and vibratory percepts are shaded in blue and teal. Participants verbally reported the intensity of each stimulus on a self-selected scale. Reported intensities were normalized by the lowest reported value x_i_, where i = 1, 2, or 3 for participants LL01, LL02, and LL03, respectively. Images of the feet were also flipped for LL01, who was a left-side amputee, to facilitate comparison between participants.

For both PNS and vibration, participants drew the location of perceived sensations on a diagram of the foot and leg and rated the intensity level on a self-selected scale (Figure 2). If a participant did not perceive a stimulus, he assigned an intensity value equal to zero. If a stimulus felt twice as strong as a prior stimulus, he would assign an intensity value double that of the prior intensity value.

### Data Collection

Participants removed their shoes and stood with their feet placed comfortably on separate force plates (AMTI OR6-6, Watertown, MA). The insoles were secured at the location where they placed their feet with tape. Force plate data were collected at 2,500 Hz. Participants were instructed to stand quietly with their arms loose by their sides during trials. The experimenter cued participants to close their eyes at the beginning of every trial and after 5 s, the stimulus was delivered for 20 s. Five seconds after the stimulus ended, the participant was directed to relax, open their eyes, and verbally comment on the trial if desired. Stimulation conditions were: PNS inducing a tactile sensation in the missing forefoot (PNS Condition 1), PNS inducing a tactile sensation at a second location in the missing foot (PNS Condition 2), intact forefoot vibration (Vibration Condition 1), intact rearfoot vibration (Vibration Condition 2), and no sensory stimulation added (baseline/control condition). All sensory stimulation conditions were presented once in a random order to form one block of trials. Participants took a seat and rested for at least 5 min between blocks. Six blocks of data from each participant were collected.

### Data Processing and Outcome Measures

Ground reaction forces were lowpass-filtered using a fourth-order Butterworth filter with a 10 Hz cutoff frequency. Forces and centers of pressures for the two feet were combined to calculate the net center of pressure (CoP). To remove differences in foot placement and initial weight distribution between trials, the average CoP location in the 3 s prior to stimulus onset was subtracted from the CoP trial data. Mediolateral values were referenced as toward the location of the amputated limb (positive) or the intact limb (negative).

The magnitude of postural adjustments was quantified in terms of CoP path length. Path length is a commonly accepted measure of stability, and shorter path lengths indicate better postural stability (Donath et al., 2012). CoP path length was calculated according to the following equation (Eq. 1), where *N* was the total number of data samples (*N* = 50,000):

(1) $$C\,o\,P\,P\,a\,t\,h\,L\,e\,n\,g\,t\,h=\sum_{i=2}^{N}\sqrt{{\left(C\,o\,P\,x_{i}-C\,o\,P\,x_{i-1}\right)}^{2}+{\left(C\,o\,P\,y_{i}-C\,o\,P\,y_{i-1}\right)}^{2}}.$$

Average CoP angle ($\bar{∠\,C\,o\,P}$) characterized the direction of whole-body leaning, and was calculated for each trial by the following equations (Eqs. 2 and 3):

(2) $$\theta_{i}=\tan^{-1}\frac{C\,o\,P\,y_{i}}{C\,o\,P\,x_{i}}$$

(3) $$\bar{∠\,C\,o\,P}=\tan^{-1}\frac{\sum_{i=1}^{N}\sin\theta_{i}}{\sum_{i=1}^{N}\cos\theta_{i}}$$

In Eqs. 2 and 3, θ_i_ is the CoP angle at sample number *i*, CoP*x*_i_, and CoP*y*_i_ are the mediolateral and anterior-posterior CoPs at sample number *i*, and *N* is the total number of data samples within a trial. Average CoP angles were examined for the first 3 s after stimulus onset as in previous work (Kavounoudias et al., 1998) and for the full 20 s of when stimulus was applied to capture early effects and the full effect of CoP movements during the trial. Throughout the manuscript, we will refer to the first 3 s as the “initial response” and to 20 s as the “full response.”

### Statistical Analyses

Changes in path length with stimulation condition for each participant were assessed via *t*-test. For each participant, a one sample *t*-test was applied to compare the path length of 12 trials of PNS (combined Conditions 1 and 2) to the mean path length of the six trials with no stimulation. A similar test was performed to compare vibration to the trials with no stimulation. These one-sample *t*-tests were one-tailed because we anticipated that path lengths would be higher in conditions with either PNS or vibration. Paired *t*-tests directly compared PNS to vibration for each participant. The 12 trials of PNS were compared to the 12 trials of vibration in each two-tailed *t*-test. This test was two-tailed because we did not have an *a priori* indication about which stimulus would have a stronger effect on path length. A Bonferroni correction was applied by adjusting the significance level to account for multiple path length comparisons within each participant’s data (*p* ≤ α/3).

Direct comparisons of CoP angles with PNS to those with vibration were made with Watson-Williams two-sample tests for each participant’s responses (Watson and Williams, 1956; Stephens, 1969). The mean CoP angles for vibration were mirrored across the vertical axis so that they could be directly compared to PNS without distinguishing between left and right foot.

Under each condition, a v-test determined whether CoP angles were randomly distributed over a circle or had a significant tendency to cluster around the predicted CoP angle (Zar, 1999). CoP angle predictions were based on previous observations indicating that CoP shifted in the direction opposite of the applied stimulus (Kavounoudias et al., 1998). For example, if the rearfoot of the right foot was vibrated, a lean forward and to the left would be predicted.

Statistical analyses were performed in MATLAB using the Statistics and Machine Learning Toolbox (*t*-tests) and the circular statistics toolbox (v-tests and Watson-Williams tests) (Berens, 2020). In all analyses, significance levels of α = 0.05 defined a statistically significant result.

## Results

### Perceived Sensations

Both PNS and vibration elicited repeatable perceptions of sensations in localized regions of the foot sole with similar intensities between conditions (Figure 2). In PNS Condition 1, all participants felt sensations on their forefoot. In PNS Condition 2, LL01 felt sensation in the forefoot and rearfoot, LL02 felt sensation in the lateral midfoot, and LL03 felt sensation in the rearfoot and medial midfoot. In vibration conditions, participants felt the vibration localized to either their forefoot (Vibration Condition 1) or their rearfoot (Vibration Condition 2). LL01 and LL03 perceived the vibration in both conditions as having the same intensity, while LL02 reported that the rearfoot vibration felt slightly stronger than the forefoot vibration.

### CoP Path Length

**FIGURE 3:**
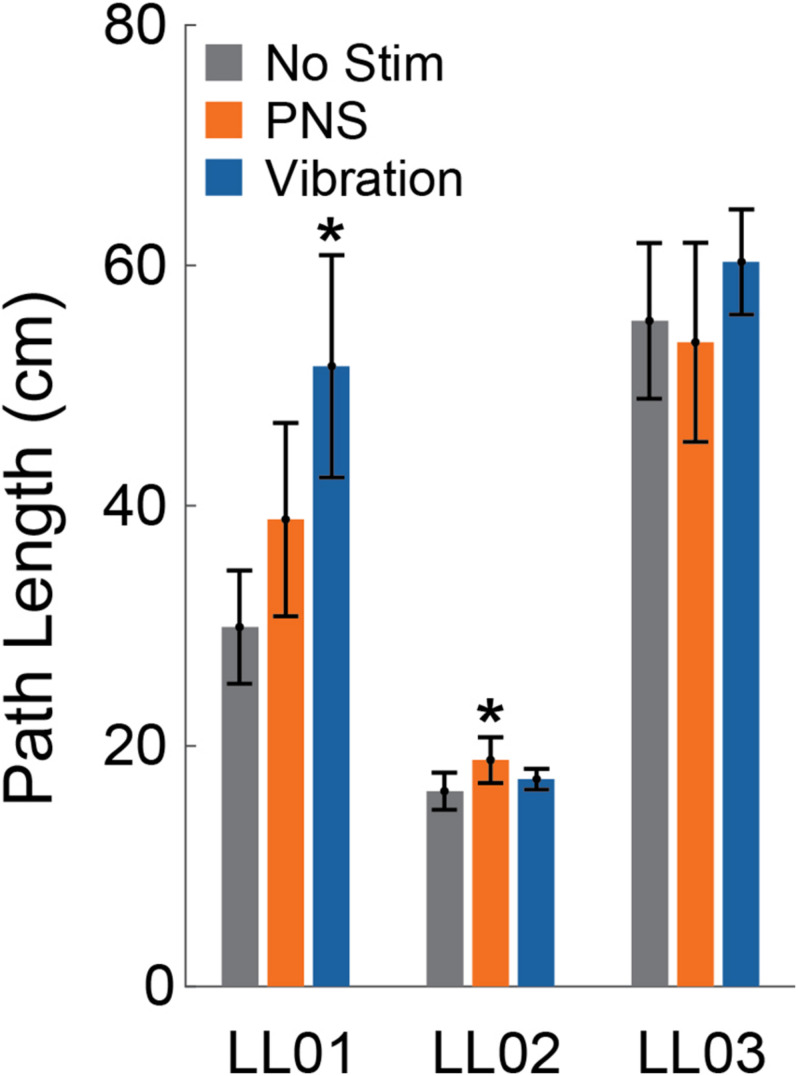
Path length. The center of pressure path length over the 20 s stimulus was averaged across trials for the control condition (No Stim, gray, *n* = 6), both peripheral nerve stimulation conditions (PNS, orange, *n* = 12), and both vibration conditions (blue, *n* = 12) for each participant (LL01, LL02, LL03). Error bars indicate 95% confidence intervals and * indicates that the tactile stimulus was significantly different than the no stimulation condition (*t*-test, *p* < 0.05).

**FIGURE 4:**
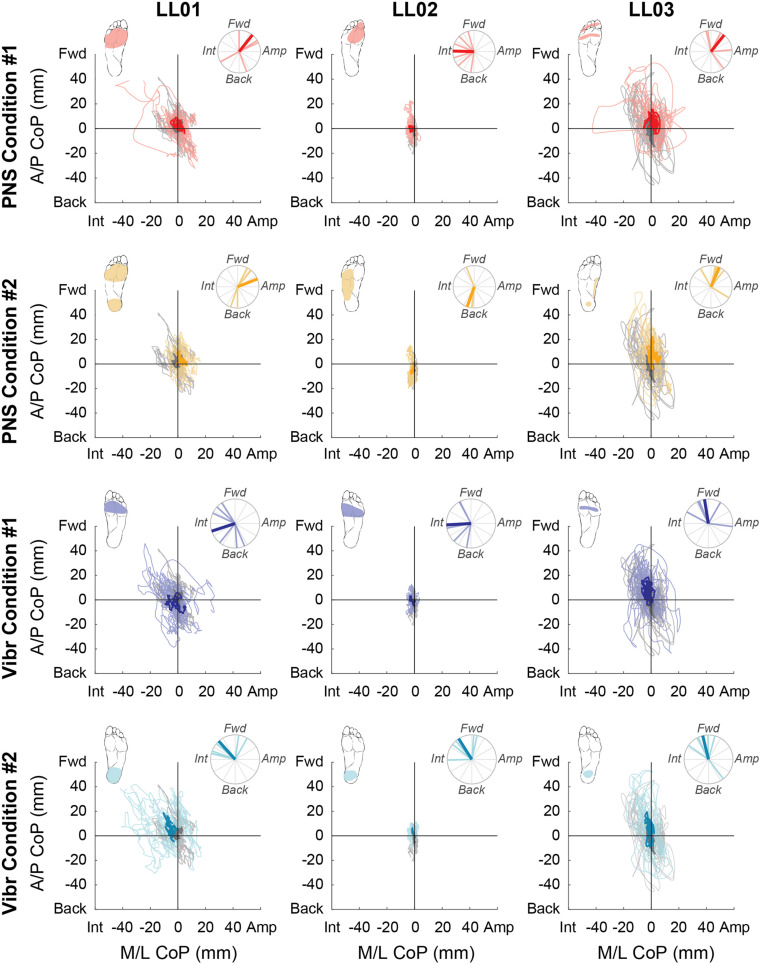
Center of pressure locations over the full 20 s trial. Tactile stimuli were delivered for 20 s with at least 3 s recorded before and after application. Positive mediolateral center of pressure (M/L CoP) values were in the direction of the amputated limb (Amp) and negative values were in the direction of the intact limb (Int). Positive anterior-posterior center of pressure (A/P CoP) values were in the forward direction (Fwd) and negative values were in the backward direction (Back). The mean CoP location across multiple trials is depicted with a bold line and the lighter traces depict CoP locations in individual trials. The center of pressure location for the no stimulation condition (gray) is shown on each plot for comparison against the locations for both peripheral nerve stimulation (PNS Condition 1, red; PNS Condition 2 yellow) and vibration conditions (Vibr Condition 1, blue; Vibr Condition 2, teal) for each participant (LL01, LL02, LL03). The perceived location of each stimulus is shown in the top left corner of each graph, while the angle of the center of pressure over the course of the whole trial is shown in the top right, where the bold line again indicates the mean CoP angle while the lighter lines indicate angles in individual trials.

Compared to trials without added stimulation, the path length was significantly larger for LL01 when receiving vibration and for LL02 when receiving PNS (one-sample *t*-test, *p* = 0.002 for LL01, *p* = 0.04 for LL02; Figure 3); other comparisons did not rise to the level of significance. Changes in path length when PNS or vibration was applied arose mostly from slowly growing shifts in the CoP location over the course of the full 20 s trial for LL02, but were a combination of increases in CoP variability and shifts in CoP location for LL01 and LL03 (Figure 4). Across all conditions, LL01 and LL03 exhibited greater variability in their CoP location than LL02 (Figure 4), which is also observable in their respective average path lengths (Figure 3).

### Comparison of CoP Locations in Initial and Full Response

**FIGURE 5:**
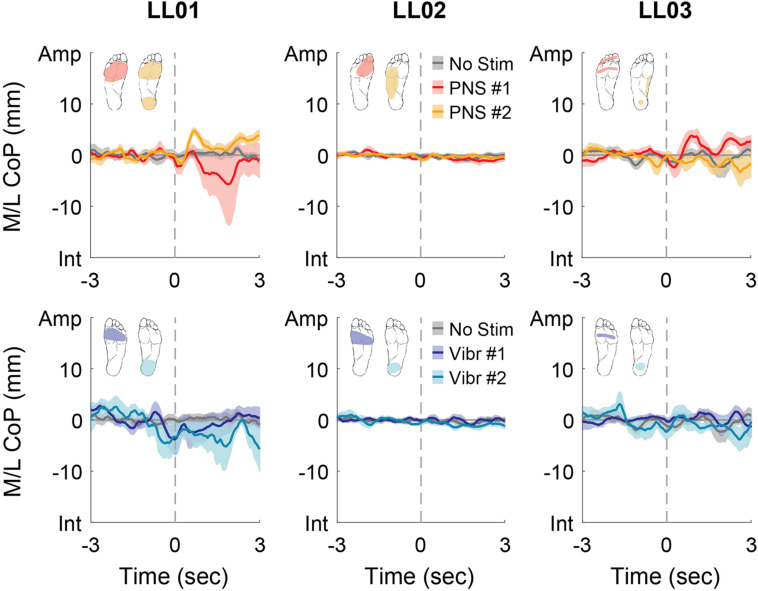
Mediolateral CoP location initial responses. The initial responses (first 3 s) to tactile stimuli were averaged across six trials. The gray dashed line indicates the onset of the stimulus. Positive mediolateral center of pressure (M/L CoP) values were in the direction of the amputated limb (Amp) and negative values were in the direction of the intact limb (Int). The mean CoP across multiple trials is depicted with a solid line and the shaded regions around each line show the standard error. The center of pressure location for the control condition (No Stim, gray) is shown on each plot for comparison against the locations for both peripheral nerve stimulation conditions (PNS #1, red; PNS #2, yellow) and both vibration conditions (Vibr #1, blue; Vibr #2, teal) for each participant (LL01, LL02, LL03).

**FIGURE 6:**
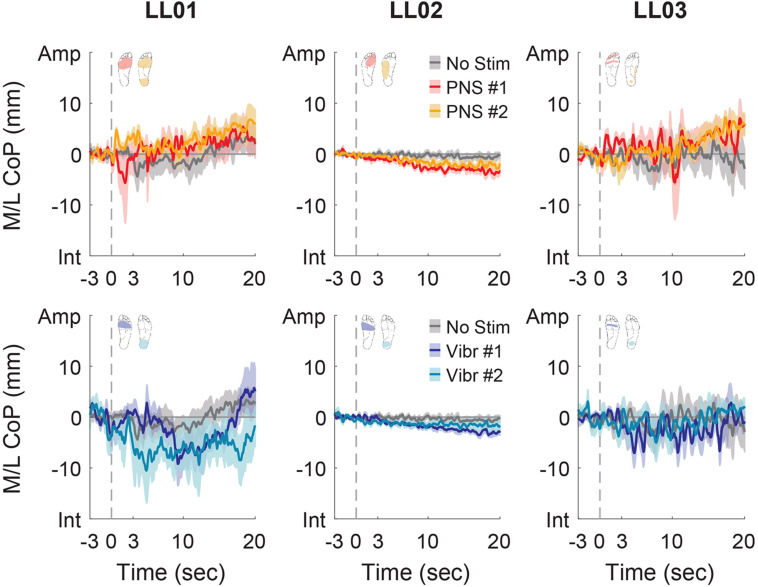
Mediolateral CoP location full responses. The responses to tactile stimuli in the full 20 s trial were averaged across six trials. The gray dashed line indicates the onset of the stimulus. Positive mediolateral center of pressure (M/L CoP) values were in the direction of the amputated limb (Amp) and negative values were in the direction of the intact limb (Int). The mean CoP across multiple trials is depicted with a solid line and the shaded regions around each line show the standard error. The center of pressure location for the control condition (No Stim, gray) is shown on each plot for comparison against the locations for both peripheral nerve stimulation conditions (PNS #1, red; PNS #2, yellow) and both vibration conditions (Vibr #1, blue; Vibr #2, teal) for each participant (LL01, LL02, LL03).

Participants made initial postural adjustments then altered their response over the course of the full trial. On average, the mean CoP angle changed significantly from the initial response to the full response for vibratory (Δ = 56°) and PNS-elicited (Δ = 41°) stimuli (paired *t*-test, *p* < 0.001). In the mediolateral direction, LL01 and LL03 had variable initial responses in the direction that they shifted their CoP (Figure 5), but over the course of the full trial they reliably shifted toward the stimulated limb (Figures 4, 6). LL02 did not have an initial response to stimulation that was noticeably different from the no stimulation condition (Figure 5), but over the course of the full trial shifted toward the intact limb for all stimulation conditions (Figures 4, 6).

**FIGURE 7:**
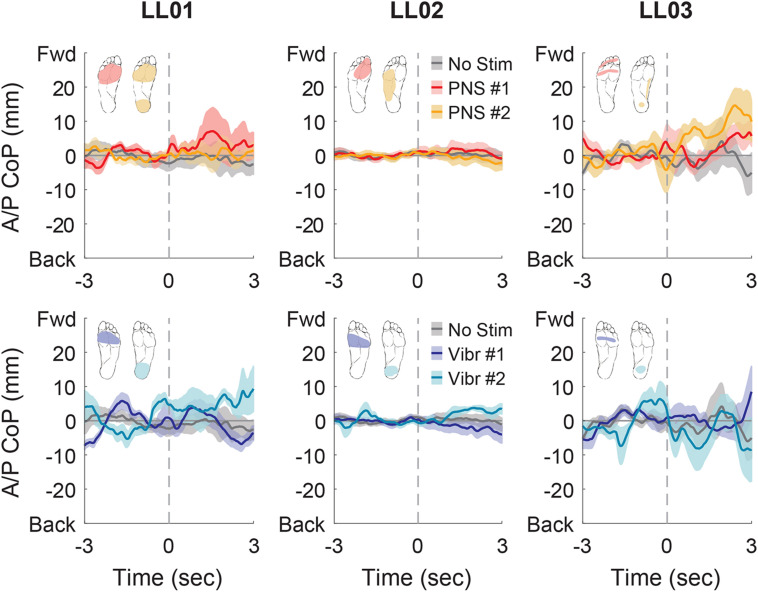
Anterior-posterior CoP location initial responses. The initial responses (first 3 s) to tactile stimuli were averaged across six trials. The gray dashed line indicates the onset of the stimulus. Positive anterior-posterior center of pressure (A/P CoP) values were in the forward direction (Fwd) and negative values were in the backward direction (Back). The mean CoP across multiple trials is depicted with a solid line and the shaded regions around each line show the standard error. The center of pressure location for the control condition (No Stim, gray) is shown on each plot for comparison against the locations for both peripheral nerve stimulation conditions (PNS #1, red; PNS #2, yellow) and both vibration conditions (Vibr #1, blue; Vibr #2, teal) for each participant (LL01, LL02, LL03).

**FIGURE 8:**
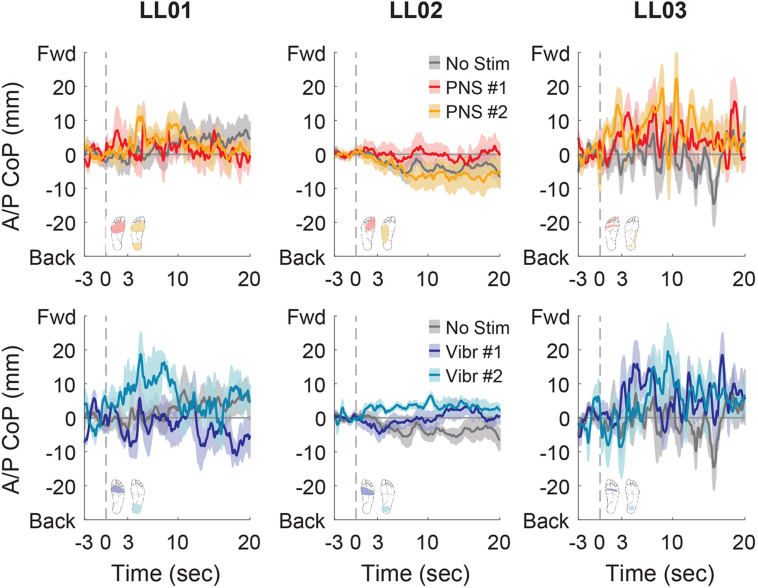
Anterior-posterior CoP location full responses. The responses to tactile stimuli in the full 20 s trial were averaged across six trials. The gray dashed line indicates the onset of the stimulus. Positive anterior-posterior center of pressure (A/P CoP) values were in the forward direction (Fwd) and negative values were in the backward direction (Back). The mean CoP across multiple trials is depicted with a solid line and the shaded regions around each line show the standard error. The center of pressure location for the control condition (No Stim, gray) is shown on each plot for comparison against the locations for both peripheral nerve stimulation conditions (PNS #1, red; PNS #2, yellow) and both vibration conditions (Vibr #1, blue; Vibr #2, teal) for each participant (LL01, LL02, LL03).

In the anterior-posterior direction, all participants initially shifted forward with PNS on the forefoot while they did not shift or shifted backwards with vibration on the forefoot (Figure 7). PNS condition 2 elicited sensation in different locations for each participant and led to different shifts: LL01 felt sensation on both the rearfoot and forefoot and did not shift, LL02 felt sensation on the side of his foot and shifted backward, and LL03 felt sensation on the rearfoot and shifted forward (Figure 7). With vibration on the rearfoot, LL01 and LL02 initially shifted forward while LL03 exhibited an anterior-posterior CoP shift similar to his shift in the control condition (Figure 7). Over the course of the full trial, LL03 leaned forward in response to all conditions with additional sensory inputs (Figures 4, 8). LL01 also leaned forward over the course of the whole trial for both PNS conditions and vibration on the rearfoot (Figures 4, 8). LL02 leaned backward over time without stimulation, but adopted a more neutral posture with PNS or vibration stimulation on the forefoot (Figures 4, 8). LL02 leaned backwards with PNS stimulation on the side of his foot and leaned forward with vibration on the rearfoot, maintaining the shift from his initial response in both cases (Figures 4, 8).

### CoP Angles Over the Full Trial

For PNS conditions, CoP shifted forward when the perceived sensations were concentrated in one area of the foot (PNS Condition 1, LL01 and LL03; PNS Condition 2 for LL03; Figure 4). When perceived sensations were distributed across the whole foot rather than concentrated in the forefoot or rearfoot (PNS Condition 2, LL01 and LL02), CoP shift in the anterior-posterior direction varied between forward and backward depending on the trial (Figure 4). Responses in the mediolateral direction were more mixed. LL01 leaned toward his amputated limb in both PNS Conditions, LL02 leaned toward his intact limb for PNS Condition 1 with no appreciable lean in PNS Condition 2, and LL03 leaned toward his amputated limb in PNS Condition 1 but showed no appreciable lean in PNS Condition 2 (Figure 4).

For vibration conditions, two of three participants (LL01 and LL02) shifted away from the area receiving vibration in the anterior-posterior direction under both conditions (Figure 4). LL03 also shifted away from the area receiving vibration in Vibration Condition 2 (Figure 4). In the mediolateral direction, two of the three participants (LL01 and LL02) shifted toward the intact leg, which was receiving stimulation.

Participants reported sensations in similar regions of the missing foot for PNS Condition 1 only. These sensations were in an area similar to the forefoot vibration condition (Figure 2). All three participants exhibited CoP angles over the full response to PNS Condition 1 that were not significantly different than those caused by vibration to the forefoot (Figure 4, Watson-Williams test, *p* > 0.05).

Overall, the average CoP angle during the initial response was only similar to that reported for able-bodied participants (Kavounoudias et al., 1998) in two cases: LL02 in Vibration Condition 1 (v-test compared to −45° *p* = 0.047) and LL03 in PNS Condition 2 (v-test compared to 135° *p* = 0.039). In all other conditions, the mean CoP angle during the initial response to a vibratory stimulus or PNS did not align significantly with the CoP angle that would be predicted based on previous work (v-test, *p* > 0.05).

## Discussion

In this study, we compared responses to sensory perturbations during standing balance with the eyes closed in three transtibial amputees using electrically elicited tactile sensations in the missing foot and vibration under the intact foot. This experiment explores whether the motor control system treats sensory inputs from PNS similarly to native tactile inputs. In addition, it demonstrates the usefulness of PNS as a novel way to provide somatosensory perturbations during tasks without interfering with the physical environment or directly modifying biomechanics. Previous work has shown that contributions of somatosensory inputs are more apparent when other sensory inputs are compromised (Buckley et al., 2002; Claret et al., 2019) and that vision can override inputs from other sources (Vanicek et al., 2009; Barnett et al., 2013). Therefore, we conducted this test without vision to maximize the impact of somatosensory perturbations on stance. Both types of sensory inputs applied in this study provided internal perturbations to standing balance, as shown by the changes in the CoP path length and directional shifts. Initial responses sometimes differed from the response over the course of the full trial, indicating that participants adapted their response the longer they were exposed to the constant sensory input.

Compared to a control condition without added sensory inputs, when either PNS or vibration was applied, path length increased. Although only significant in two instances, path length increased with PNS for two out of three participants and all participants exhibited greater CoP path lengths when vibration was applied. In general, changes in path length arose from subtle shifts in CoP location combined with increased variability over the course of a trial with altered sensory inputs. It is possible that differences in responses to vibratory and PNS inputs observed within subjects were influenced by small variations in intensity, location, and touch modality between these two conditions. However, we attempted to match perceived locations and intensities between vibration and PNS conditions, and the small differences that were present did not appear to systematically affect the magnitude of postural adjustments. The CoP path length for participant LL01 tended to be longer for vibratory stimuli than for PNS, despite his verbal report that sensations elicited by PNS felt slightly stronger (i.e., more likely to perturb balance) than vibrations. CoP path length was higher with PNS compared to control trials for LL02, with responses to vibratory stimuli falling somewhere in between, despite his verbally reporting similar intensities for both stimulus conditions. LL03 also verbally reported similar intensities between vibratory and PNS sensory inputs, but path length tended to be longer for vibration. Finally, despite our attempts to match perceived locations and sensations among participants, some differences remained. These differences arise from variations in sensations accessible by PNS due to participant-specific nerve cuff placements and distribution of neural fibers within the nerve, as well as the subjective nature of perception. Thus, some of the inter-subject variability may be explained in part by the variation between perceived locations and touch modality, in addition to differences in subject characteristics, such as age.

Previous work in which vibration was applied to the foot soles of able-bodied participants during quiet stance resulted in their leaning orthogonally away from applied vibration (e.g., vibration on the left forefoot caused participants to lean toward the right rearfoot) (Kavounoudias et al., 1998). We found that our amputee participants behaved similarly with their initial response in the anterior-posterior direction in some cases (LL01 during PNS Condition 2 and Vibration Condition 2, LL02 during both vibration conditions, LL03 during PNS Condition 2) but not in others. However, they generally moved in the opposite direction, *toward* the stimulus source, in the mediolateral direction. This was true in both the initial response and the response over the full trial. Previous investigations of CoP shifts in response to vibrational stimuli in able-bodied participants used a 100 Hz vibration frequency, while we used a 190–220 Hz vibration frequency. Vibrations between 70 and 115 Hz induce strong illusions of muscle movement sensations (Goodwin et al., 1972; Roll and Vedel, 1982; Marasco et al., 2017). The strength and incidence of movement illusions decreases rapidly as the vibration frequency departs from this range (Marasco et al., 2011, 2017). Furthermore, muscle spindles, which help detect muscle movement, respond optimally to 20–100 Hz frequencies (Burke et al., 1976; Pyykkö et al., 1989), while Pacinian corpuscles, which detect vibration, respond best to 150–270 Hz frequencies (Sato, 1961; Bolanowski and Zwislocki, 1984; Gescheider et al., 2001). It is likely that the differences in vibratory stimuli between our experiment and previous work affected different receptors, which lead to different sensory feedback (tactile instead of proprioceptive). Thus, the vibration that we applied in our study would not be expected to induce illusions of movement, but rather to disrupt natural somatosensation by changing the tactile feedback, leading to different CoP location shifts. Likewise, the participants did not report that electrically elicited sensations resulted in movement illusions; rather, the sensations felt like tingling, pulsating, or pressure from toes curling. It is possible that both our vibration and PNS results differ from previous work due to the modality, or quality, of the tactile stimuli.

It is also possible that amputees respond differently to unilateral tactile stimuli than able-bodied participants. During standing, amputees commonly load their intact leg more than their amputated leg (for review, Ku et al., 2014). Previous work has also shown postural shifts away from regions of insensitivity (for review, Li et al., 2019), which would also encourage shifts toward the intact leg. Across all trials with stimulation, we found that application on the intact side caused ipsilateral shifts in 50% of the trials while stimulation on the missing foot caused ipsilateral shifts in 33% of the trials. A predisposition toward loading on the intact side could have caused some of the differences from previously observed mediolateral CoP directional shifts in able-bodied participants.

The ability of PNS to elicit postural adjustments suggests that input from PNS is incorporated into the sensorimotor control scheme. Our participants were experienced with sensations elicited by PNS, which allowed them to develop strategies for incorporating it into their decision-making processes. If participants had been unfamiliar with PNS, the results would have been confounded by any transient period spent updating the way that somatosensory sensations are incorporated into motor control decisions. Sensory information is incorporated from multiple sources and relied upon differently depending on the nature and variability of the feedback (Oie et al., 2002; Bays and Wolpert, 2007). Even though amputees typically adjust to relying on information from only the intact limb and proximal regions of the residual limb, our results show that participants can incorporate information from PNS over the course of a few seconds, similar to native tactile feedback. Furthermore, PNS perceived as arising from locations similar to those of vibration stimuli caused similar directional shifts, indicating that the internal model treats perturbations “elicited” by PNS similarly to perturbations “detected” by mechanoreceptors in the intact foot sole. This adds to the growing body of evidence that PNS provides useful information for balance control and movement planning (Petrini et al., 2019a; Charkhkar et al., 2020; Christie et al., 2020).

Our results provide evidence that PNS can act as an internal perturbation separate from environmental conditions (i.e., when the support surface and surroundings are unchanged). Our approach also facilitates clear separation of tactile from proprioceptive feedback and portable artificial alteration of tactile sensation without modifying user biomechanics *a priori* (e.g., through items inserted in the shoe). Selective manipulation of the PNS sensory inputs could be used to study the response of the internal model to injected noise during a task (such as feedback from an incorrect location or with a time delay). Future work would first need to more rigorously explore internal model strength and uncertainty with and without PNS tactile feedback, for example with a psychophysical adaptation test to measure modifications in control strategy based on feedback (Engels et al., 2019).

This study was limited by a small sample size due to the low number of lower-limb amputees who have received the PNS system. Despite a thorough sweep of PNS parameters, participants available for this study reported sensations in other parts of the foot or residual limb along with elicited sensation in the rearfoot. Thus, we were unable to consistently isolate sensations in the rearfoot with monopolar stimulation. Other stimulation options, such as steering the electric field toward other sensory fibers representing the rearfoot with currents from multiple contacts, could be explored in the future. In order to prevent participant fatigue during a session and avoid introducing additional variation from testing in multiple sessions while still collecting sufficient repetitions for each condition, we repeated each condition six times within a single session. We chose this number of repetitions based on the number of conditions we planned to compare and previous observations of quiet stance postural adjustments in response to vibration (Kavounoudias et al., 1998). Our analyses revealed some significant differences between conditions, and *post-hoc* power analyses show that additional differences in our data would remain below the level of significance unless far more repetitions were performed than could be completed in even three sessions. Thus, six repetitions suitably balanced study design factors in this exploration of our planned hypotheses.

In this work, we focused exclusively on tactile sensory perturbations in a single, simple task with limited environmental distractions. Stimulation from the PNS system can also provide sensations of muscle tightening and joint movement (Charkhkar et al., 2018). Future work should compare the effects of proprioceptive sensations induced in the intact limb (e.g., through TENS-unit-induced muscle tightening or movement illusions) with those induced in the missing limb by PNS. We also plan to explore the effects of somatosensory perturbations created using our method in other balance and locomotion conditions, such as overground walking, obstacle crossing, and walking on uneven ground. In addition to studying changes in ground reaction forces and biomechanics, future work could also explore the effects on muscle activation through electromyography and on central nervous system activity through electroencephalography.

Unilateral transtibial amputees adjusted their posture in response to PNS-induced tactile sensations perceived as coming from the missing foot sole. These responses demonstrated that the sensorimotor control system reacts to PNS-induced tactile sensation and that PNS can induce internal perturbations. Similarities between responses to PNS and responses to vibration on the intact foot sole indicate that the sensorimotor system treats tactile inputs induced by PNS similarly to native tactile inputs. PNS sensory inputs can provide information about foot-ground contact by modulating PNS in response to readings from pressure sensors placed underneath the prosthetic foot (Charkhkar et al., 2020; Christie et al., 2020). Our observations encourage future investigation of the dynamics of internal model responses to PNS feedback, such as threshold detection of changes during stance (e.g., acceleration akin to a slip, Richerson et al., 2003) and adaptation during tasks with and without feedback (e.g., Engels et al., 2019). Our findings have implications beyond restoration of foot sole tactile feedback to impaired populations. Since PNS can act as an internal perturbation to tactile feedback used in sensorimotor control, it could also be used to investigate the robustness of motor control to real-time manipulations of tactile feedback. These manipulations could even directly conflict with reality, such as providing sensations of toe-off at heel-strike. This would represent a novel experimental paradigm to separate proprioceptive feedback from tactile feedback. It would also supply an alternative method to investigate relative reliance on the different sensory modalities in a variety of conditions to expand our knowledge and understanding of the sensorimotor system.

## Data Availability Statement

The datasets for this article are not publicly available because of restrictions in the IRB-approved protocol. Requests to access the datasets should be directed to RT, ronald.triolo@case.edu.

## Ethics Statement

This study involved human participants and was reviewed and approved by the Louis Stokes Cleveland Veterans Affairs Medical Center Institutional Review Board and Department of the Navy Human Research Protection Program. Participants provided their written informed consent to take part in this study.

## Author Contributions

CS conceived the study design. CS, BC, and HC conducted the experiments and initial discussion of the results and manuscript content. BC and CS performed the data analysis. CS drafted the manuscript with contributions from BC. HC and RT supervised the project. All authors contributed to design development, data interpretation, and manuscript editing.

## Conflict of Interest

The authors declare that the research was conducted in the absence of any commercial or financial relationships that could be construed as a potential conflict of interest.
