# Supplementary material for: Lower-Limb Amputees Adjust Quiet Stance in Response to Manipulations of Plantar Sensation
Source: Front Neurosci. 2021 Feb 18;15:611926. doi: 10.3389/fnins.2021.611926 (PMC7930749; doi:10.3389/fnins.2021.611926)
Supplement: Supplementary file 1 [file Table_1.docx]

Supplementary Material

# Supplementary Figures and Tables

## Supplementary Table

**Table 1.** Peripheral nerve stimulation parameters used to elicit sensations in each of the three participants (LL01, LL02, LL03) for the peripheral nerve stimulation (PNS) conditions. Contacts that elicited sensations on the foot sole were chosen (e.g., S2: sciatic cuff, contact 2). Multiple contacts elicited sensations on the foot sole, but the ones chosen were those for which pulse amplitude (PA), pulse width (PW), and pulse frequency (PF) could be tuned to elicit tactile sensations on the foot sole that were most similar in regions and intensity to sensations reported by the participants when the vibratory stimuli were applied.

|  | PNS Condition #1 | | | | PNS Condition #2 | | | |
| --- | --- | --- | --- | --- | --- | --- | --- | --- |
|  | **Contact** | **PA (mA)** | **PW (μs)** | **PF (Hz)** | **Contact** | **PA (mA)** | **PW (μs)** | **PF (Hz)** |
| **LL01** | S2 | 1.2 | 240 | 20 | DS3 | 1.2 | 200 | 100 |
| **LL02** | G8 | 1.1 | 180 | 20 | G4 | 0.8 | 180 | 20 |
| **LL03** | PT1 | 0.9 | 120 | 20 | PT8 | 1.2 | 200 | 20 |
